# Supplementary material for: Innate lymphocyte-induced CXCR3B-mediated melanocyte apoptosis is a potential initiator of T-cell autoreactivity in vitiligo
Source: Nat Commun. 2019 May 16;10:2178. doi: 10.1038/s41467-019-09963-8 (PMC6522502; doi:10.1038/s41467-019-09963-8)
Supplement: Supplementary file 1 — Supplementary Information [file 41467_2019_9963_MOESM1_ESM.pdf]

Supplementary Information for Manuscript NCOMMS-18-29842A

Title: Innate lymphocyte-induced CXCR3B-mediated melanocyte apoptosis is a potential initiator of T-cell autoreactivity in vitiligo

Author: MK Tulic et al.,

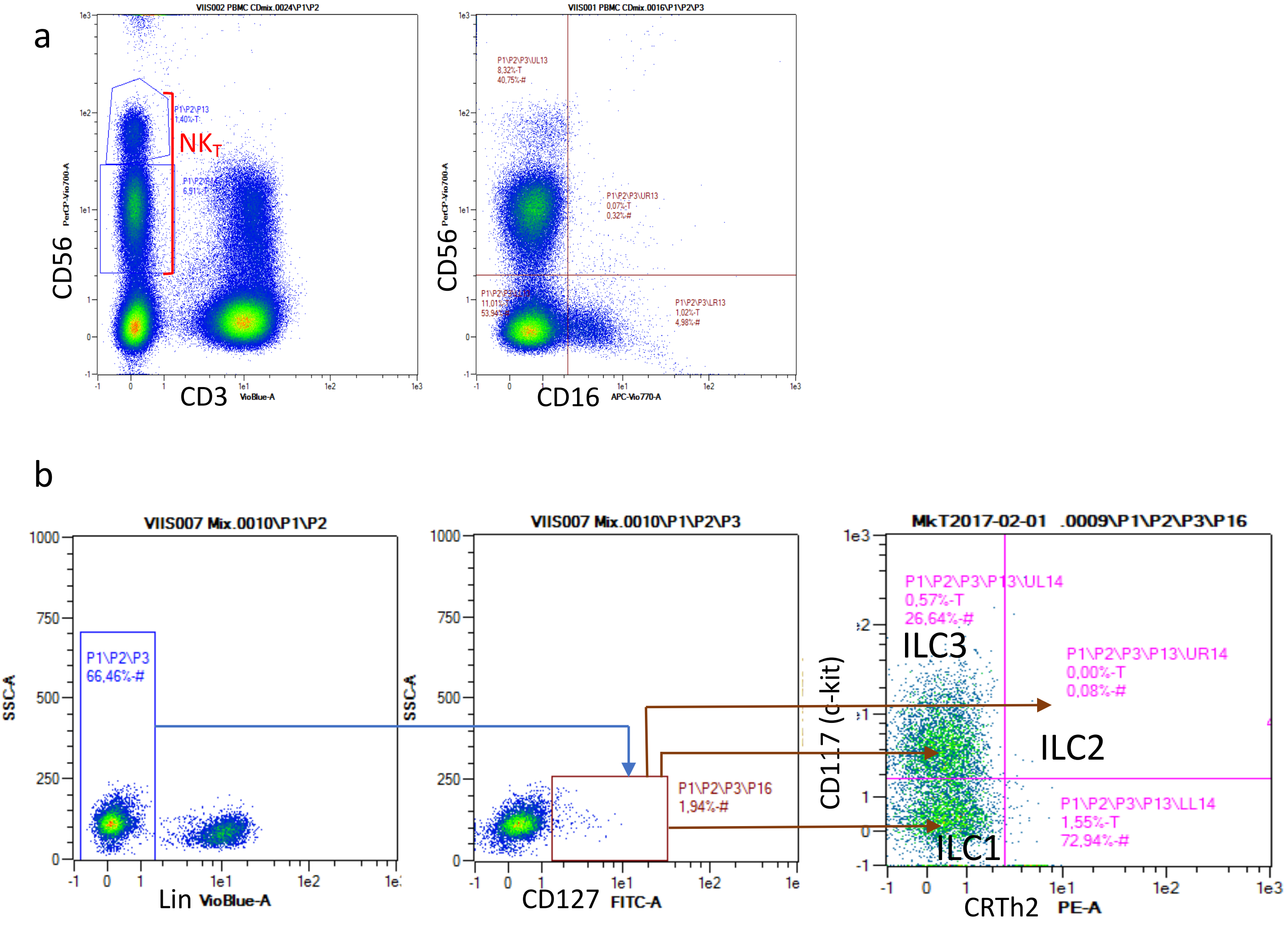

Supplementary Figure 1. Gating strategies for FACS analysis and sorting of peripheral blood NKs and ILCs

Total NKs (NK_T) were defined as CD3-CD56+ cells and cytotoxic or cytokine-producing NKs differentiated based on their additional CD16 expression ($CD56^{bright}$ $CD16^{dim}$ cells as cytokine producing and $CD56^{dim}$ $CD16^{bright}$ as cytotoxic NK cells) (a). Gating strategy for ILC subpopulations (b) involved negative Lineage selection to exclude CD3+ T cells, CD19+ B cells, CD14+ macrophages, CD34+ eosinophils, CD123+ DCs and TCR followed by CD127 positivity to exclude NKs. Finally, CD117 (c-kit) and CRTh2 (CD294) were used to delineate the 3 ILC subclasses whereby ILC1 were Lin-CD127+CRTh2-CD117-, ILC2 Lin-CD127+CRTh2+CD117+ and ILC3 Lin-CD127+CRTh2-CD117+ cells.

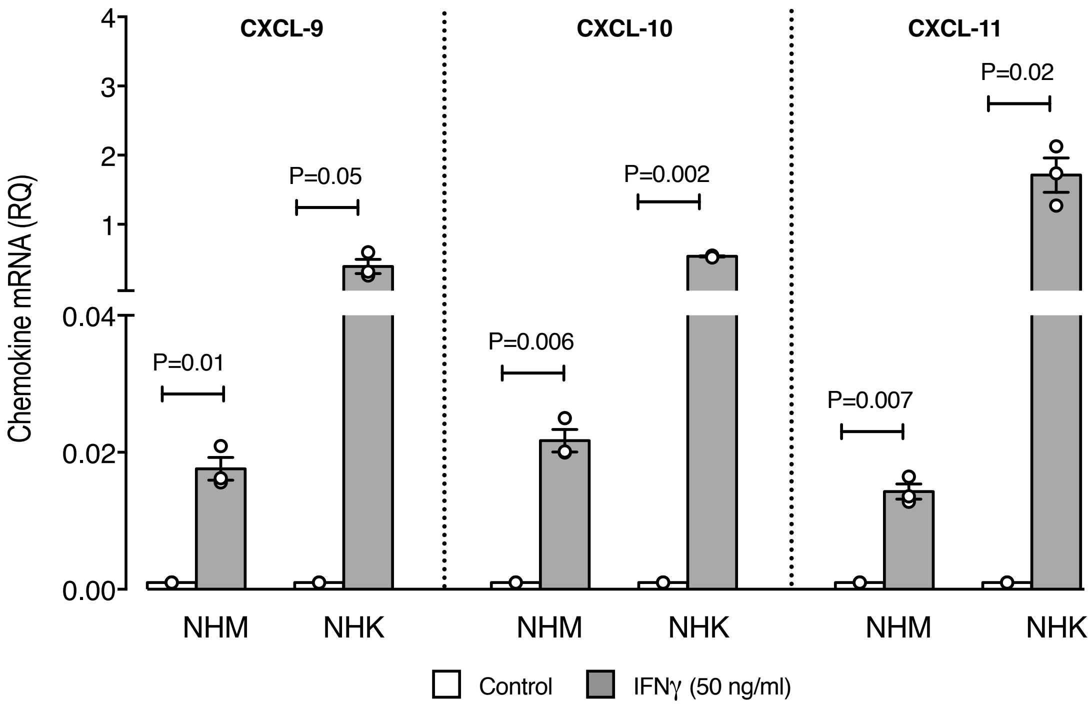

Supplementary Figure 2. Chemokine production by human melanocytes and keratinocytes

CXCL-9, CXCL-10 and CXCL-11 mRNA responses in normal human melanocytes (NHM, n=5) and normal human keratinocytes (NHK, n=6) before (white bars) and 24 hrs after stimulation with IFNg (50 ng/ml) (grey bars). Results are shown as individual dot plots of relative quantity of chemokine mRNA compared to housekeeping gene SB34, with a line at mean ± SEM.

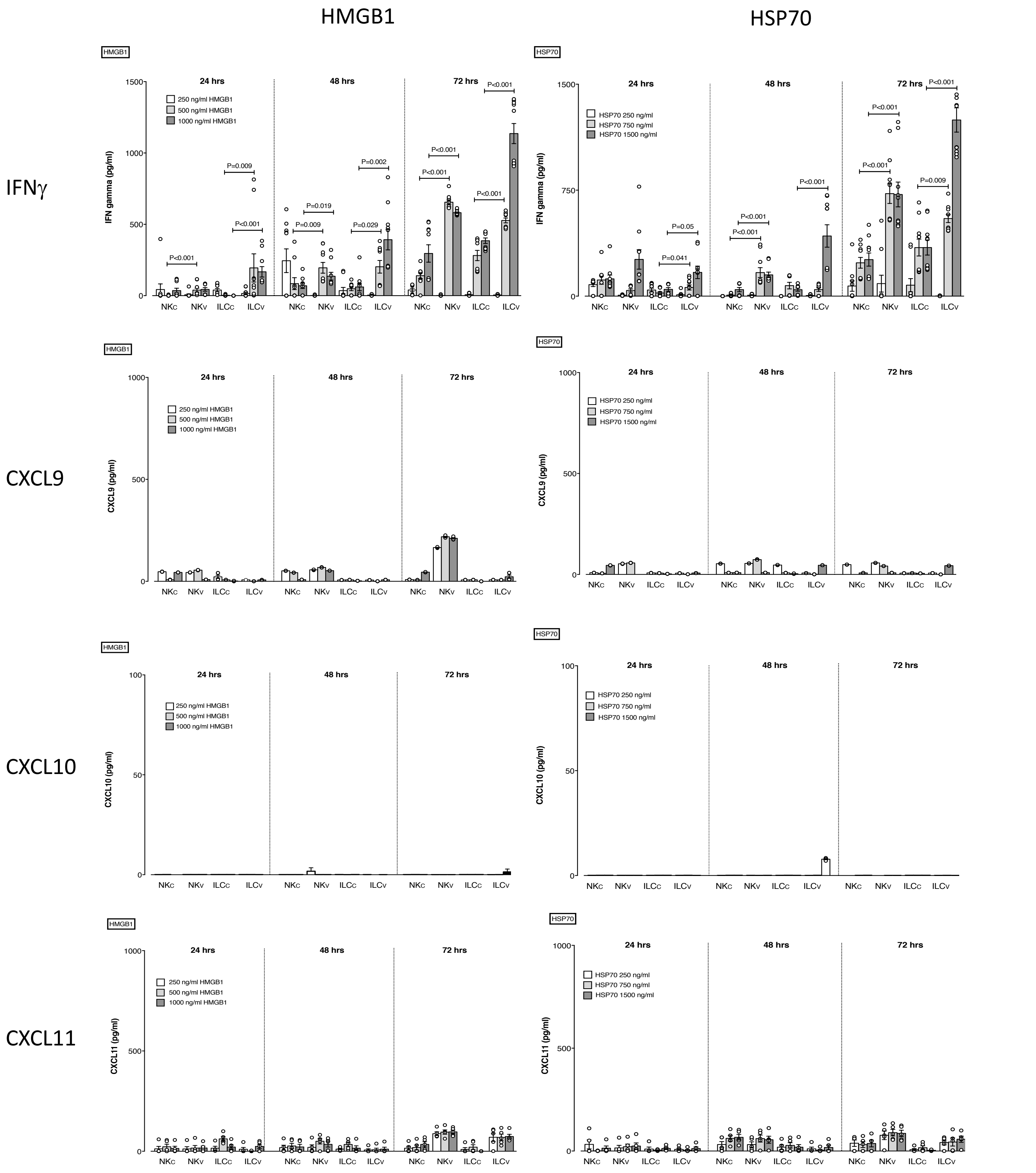

Supplementary Figure 3. Effect of DAMPs (damage associated molecular patterns) on melanocyte IFNγ and chemokine production

IFNγ, CXCL9, CXCL10 and CXCL11 production by sorted NK and ILCs from blood of healthy control (c, n=5) and vitiligo (v, n=5) subjects following *in vitro* stimulation with HMGB1 (250-1000 ng/ml) or HSP70 (TKD, 250-1500 ng/ml) for 24, 48 or 72 hrs. Results are shown as individual dot plots with a line at mean ± SEM.

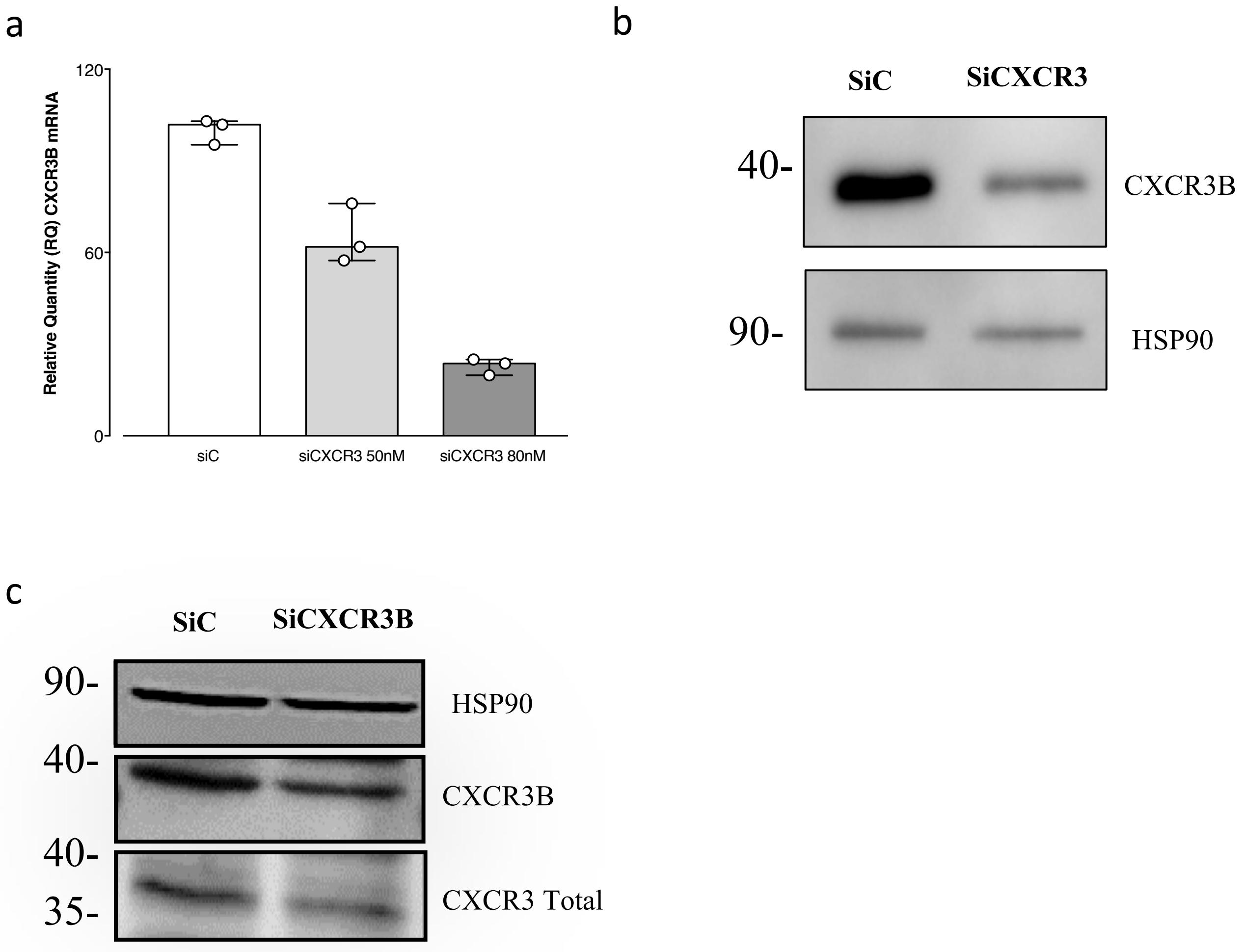

Supplementary Figure 4. Efficiency of siCXCR3 silencing

CXCR3B mRNA (a) and CXC3B protein (bc) expression in normal human primary melanocytes transfected for 72 hrs with siCXCR3 (at 50 nM or 80 nM) (ab) or siCXCR3B (c). mRNA results are shown as individual dot plots with a line at mean ± SEM. Protein data are shown as a representative WB of 3 separate experiments.

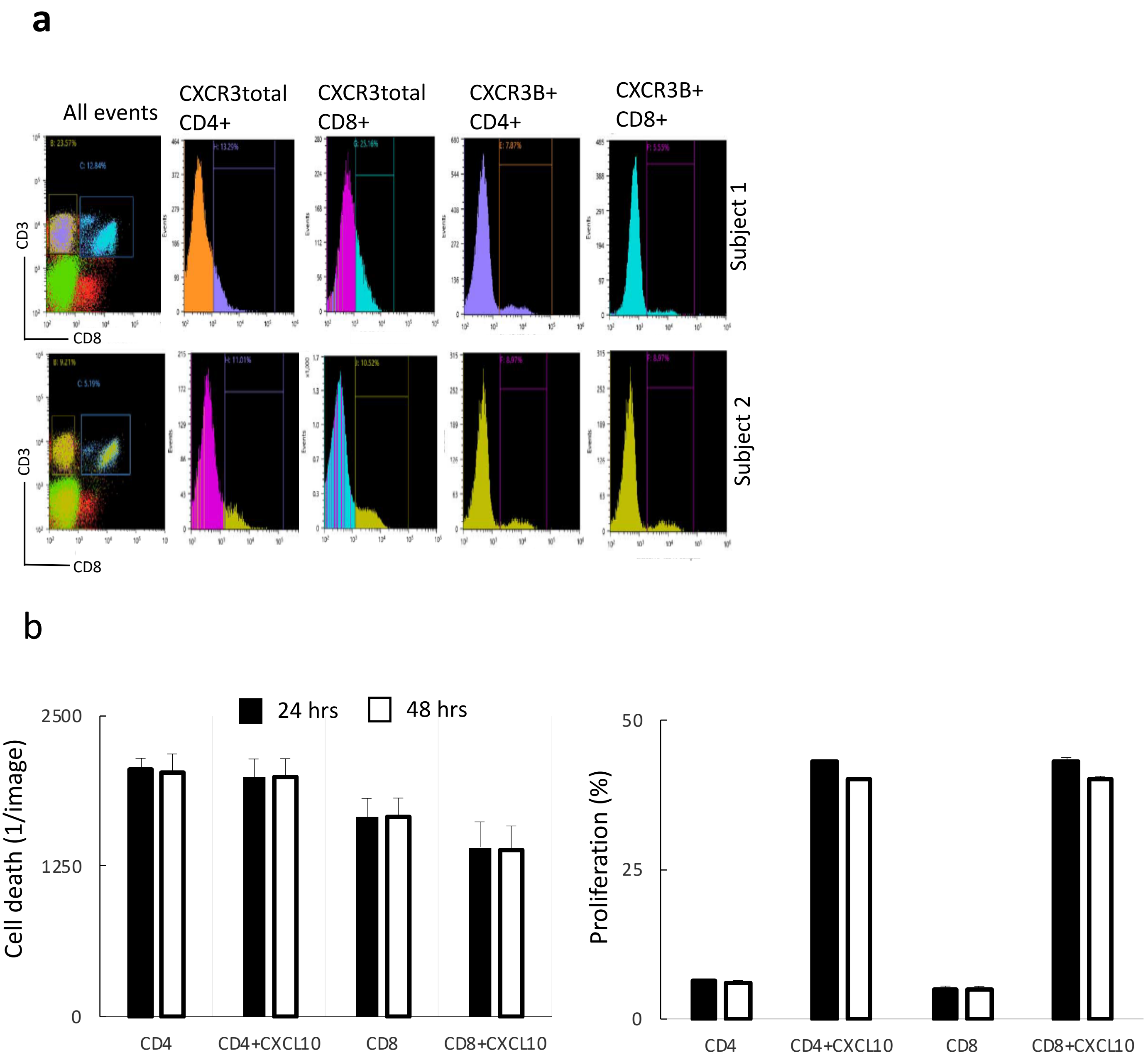

Supplementary Figure 5. Expression and function of CXCR3 in human T cells

(a) Low expression of CXCR3B on CD4+ (4th column) and CD8+ (last column) lymphocytes isolated from peripheral blood mononuclear cells (PMBCs) of 2 healthy patients. CXCL10 stimulation of CD4+ or CD8+ T cells has no effect on their death (b top) however it stimulates their proliferation at both 24 and 48 hrs (b bottom).

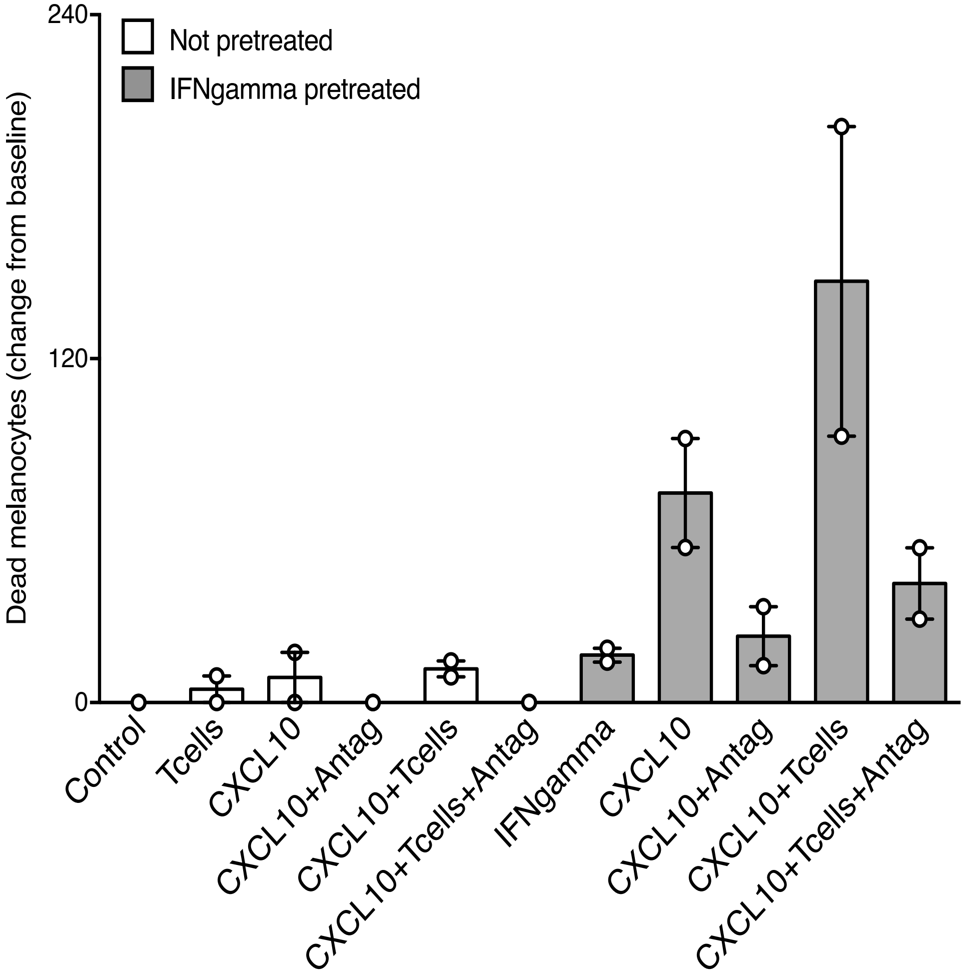

Supplementary Figure 6. Allogeneic PBMC further exacerbate CXCL10-induced melanocyte death (positive control)

Melanocytes extracted from vitiligo skin (n=2) were primed (grey bars) or not (white bars) with IFNγ (50 ng/ml) for 48 hrs prior exposure to CXCL10 (100 pg/ml) in presence or absence of CXCR3 Antagonist AS612568 (2 mM). 24 hrs later, melanocyte media was replaced and 72 hrs later allogeneic PBMCs ($1x10^6$/ml) were added to melanocytes. Melanocytes were tracked with CellTracker™ Red CMPTX dye and IncuCyte® Cytotox Green reagent added to the co-culture to track all dead cells. Cell viability was monitored using IncuCyte® live cell fluorescence imaging system. Dead melanocytes are represented as yellow co-localized cells and results are shown as individual dot plots with a line at mean ± SEM.

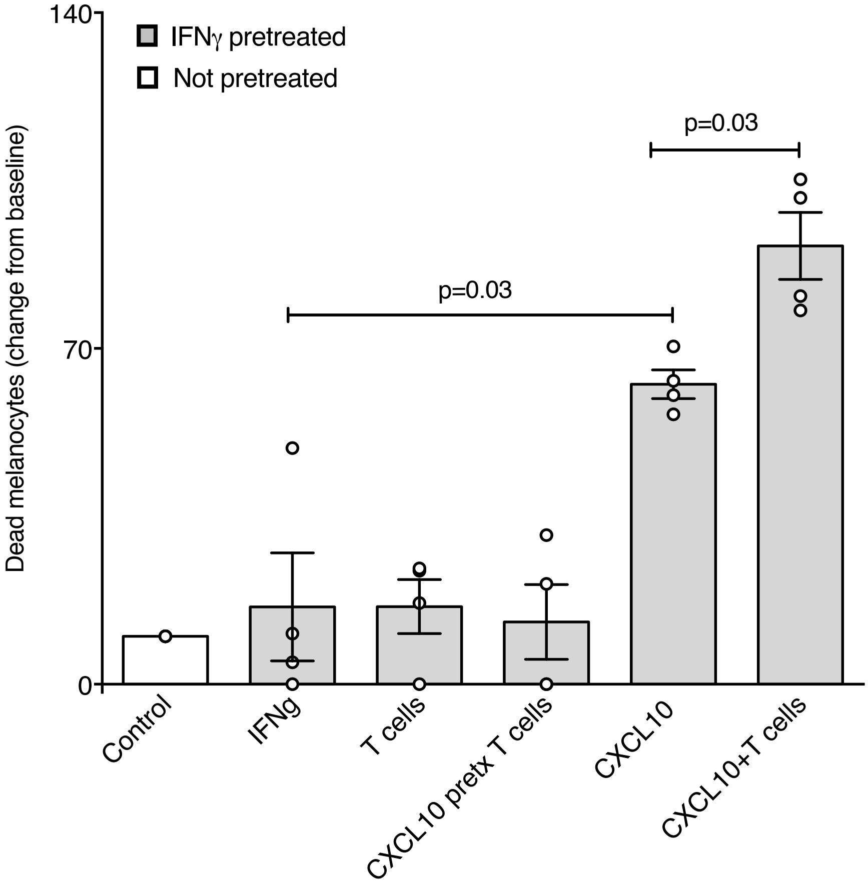

Supplementary Figure 7. CXCL10 has no direct effect on T cells

Melanocytes were primed with IFNγ (50 ng/ml) for 48 hrs (grey bars, n=4) prior to exposure to CXCL10 (100 pg/ml). 24 hrs later, media was replaced and 72 hrs later, *allogeneic* PBMCs (1x10^6/ml) were added to melanocytes. The direct effect of CXCL10 on T cells was examined by pre-treating T cells with CXCL10 for 24 hrs *prior* to placing them in co-culture with IFNγ–treated melanocytes. Cell viability was monitored using IncuCyte® live cell fluorescence imaging system. Results are shown as a change in the number of dead melanocytes from unprimed controls (white bars, n=4) and represented as individual dot plots with a line at mean ± SEM.

	Forward	Reverse	Dilution
**SB34**	TGCTCAGTACCCATTCTATCAT	AAGGTAATCCGTCTCCACAGA	1/10
**IFNg**	TCAGCCATCACTTGGATGAG	CGAGATGACTTCGAAAAGCTG	1/10
**CXCL4**	TGAAGAATGGAAGGAAAATTTGC	CAAATGCACACACGTAGGCAGCT	1/10
**CXCL9**	TCACATCTGCTGAATCTGGG	CCTTAAACAATTTGCCCCAA	1/50
**CXCL10**	GCTGATGCAGGTACAGCGT	CACCATGAATCAAACTGCGA	1/10
**CXCL11**	ATGAGTGTGAAGGGCATGGC	TCACTGCTTTTACCCCAGGG	1/10
**CXCR3 Total**	AGCCCAGCCATGGTCCTTGA	CTGTAGAGGGCTGGCAGGAA	1/10
**CXCR3B**	TGCCAGGCCTTTACACAGC	TCGGCGTCATTTAGCACTTG	1/10

Supplementary Table 1. Sequences of primers used
