# Supplementary material for: Innate lymphocyte-induced CXCR3B-mediated melanocyte apoptosis is a potential initiator of T-cell autoreactivity in vitiligo
Source: Nat Commun. 2019 May 16;10:2178. doi: 10.1038/s41467-019-09963-8 (PMC6522502; doi:10.1038/s41467-019-09963-8)
Supplement: Supplementary file 3 — Reporting Summary [file 41467_2019_9963_MOESM3_ESM.pdf]

nature research

Corresponding author(s): NCOMMS-18-29842-A

Last updated by author(s): 18/03/2019

Reporting Summary

Nature Research wishes to improve the reproducibility of the work that we publish. This form provides structure for consistency and transparency in reporting. For further information on Nature Research policies, see Authors & Referees and the Editorial Policy Checklist.

Statistics

For all statistical analyses, confirm that the following items are present in the figure legend, table legend, main text, or Methods section.

n/a	Confirmed	
☐	☒	The exact sample size (*n*) for each experimental group/condition, given as a discrete number and unit of measurement
☐	☒	A statement on whether measurements were taken from distinct samples or whether the same sample was measured repeatedly
☐	☒	The statistical test(s) used AND whether they are one- or two-sided<br>*Only common tests should be described solely by name; describe more complex techniques in the Methods section.*
☐	☒	A description of all covariates tested
☐	☒	A description of any assumptions or corrections, such as tests of normality and adjustment for multiple comparisons
☐	☒	A full description of the statistical parameters including central tendency (e.g. means) or other basic estimates (e.g. regression coefficient) AND variation (e.g. standard deviation) or associated estimates of uncertainty (e.g. confidence intervals)
☐	☒	For null hypothesis testing, the test statistic (e.g. *F*, *t*, *r*) with confidence intervals, effect sizes, degrees of freedom and *P* value noted<br>*Give P values as exact values whenever suitable.*
☒	☐	For Bayesian analysis, information on the choice of priors and Markov chain Monte Carlo settings
☒	☐	For hierarchical and complex designs, identification of the appropriate level for tests and full reporting of outcomes
☒	☐	Estimates of effect sizes (e.g. Cohen's *d*, Pearson's *r*), indicating how they were calculated

Our web collection on statistics for biologists contains articles on many of the points above.

Software and code

Policy information about availability of computer code

Data collection	N/A
Data analysis	Graphpad Prism® 6 software

For manuscripts utilizing custom algorithms or software that are central to the research but not yet described in published literature, software must be made available to editors/reviewers. We strongly encourage code deposition in a community repository (e.g. GitHub). See the Nature Research guidelines for submitting code & software for further information.

Data

Policy information about availability of data

All manuscripts must include a data availability statement. This statement should provide the following information, where applicable:

- Accession codes, unique identifiers, or web links for publicly available datasets
- A list of figures that have associated raw data
- A description of any restrictions on data availability

The data that support the findings of this study are available from the corresponding author upon reasonable request.

Field-specific reporting

Please select the one below that is the best fit for your research. If you are not sure, read the appropriate sections before making your selection.

☒ Life sciences ☐ Behavioural & social sciences ☐ Ecological, evolutionary & environmental sciences

For a reference copy of the document with all sections, see nature.com/documents/nr-reporting-summary-flat.pdf

Life sciences study design

All studies must disclose on these points even when the disclosure is negative.

Sample size	Determined by previous experiments done in the same laboratory using the same tissue/cells
Data exclusions	No data were excluded from the analyses
Replication	All experiments performed in triplicate
Randomization	N/A
Blinding	N/A

Reporting for specific materials, systems and methods

We require information from authors about some types of materials, experimental systems and methods used in many studies. Here, indicate whether each material, system or method listed is relevant to your study. If you are not sure if a list item applies to your research, read the appropriate section before selecting a response.

Materials & experimental systems

n/a	Involved in the study	
☐	☒	Antibodies
☒	☐	Eukaryotic cell lines
☒	☐	Palaeontology
☒	☐	Animals and other organisms
☐	☒	Human research participants
☒	☐	Clinical data

Methods

n/a	Involved in the study	
☒	☐	ChIP-seq
☐	☒	Flow cytometry
☒	☐	MRI-based neuroimaging

Antibodies

Antibodies used	Please see attached sheet
Validation	Each antibody was chosen based on its validation and certification of its function by the manufacturer for the application used . Once received, its effectiveness was validated and concentration of use optimized for our experiments.

Human research participants

Policy information about studies involving human research participants

Population characteristics	The total number of participants is 22.<br>14 non-segmental vitiligo (male/female ratio 7/7). Mean age 48 (range 43-56) with a disease duration lasting from 4 months to 14 years; 1 has a familial history of vitiligo and 2 had an auto-immune thyroid disease.<br>8 controls (male/female ratio 5/3). Mean age 51 (range 39-74). None of the controls had any auto-immune or inflammatory disorders. These 2 populations were well matched for age and gender distribution.
Recruitment	Participants are outpatients from the Department of Dermatology of CHU Nice, France. All the samples were obtained under strict protocols approved by the National Ethics Committee (N12.034).
Ethics oversight	Comite de Protection des Personnes (CPP), CHU Hopital de Cimiez

Note that full information on the approval of the study protocol must also be provided in the manuscript.

Flow Cytometry

Plots

Confirm that:

☒ The axis labels state the marker and fluorochrome used (e.g. CD4-FITC).

☒ The axis scales are clearly visible. Include numbers along axes only for bottom left plot of group (a 'group' is an analysis of identical markers).

☒ All plots are contour plots with outliers or pseudocolor plots.

☒ A numerical value for number of cells or percentage (with statistics) is provided.

Methodology

Sample preparation	Fresh peripheral blood mononuclear cells were isolated by Ficoll gradient, cells stained and passed through MACSQuant® Analyzer 10 Flow Cytometer. For sorting of NK and ILC populations we used Sony Cell Sorter SH800.
Instrument	MACSQuant® Analyzer 10 Flow Cytometer
Software	MACSQuantify™ Software inbuilt into MACSQuant® Analyzer 10 Flow Cytometer
Cell population abundance	Total ILC represent <1% PBMC and as a result we obtained 50 mL blood from each patient to ensure maximal retrieval of these cells to do functional assays.
Gating strategy	Total NKs were defined as CD3-CD56+ cells. Cytotoxic or cytokine-producing NKs were differentiated based on their additional CD16 expression whereby CD56bright CD16dim cells are cytokine producing NKs and CD56dim CD16bright are cytotoxic NK cells. Gating strategy for ILC subpopulations initially involved gating all live PBMCs for negative Lineage selection (to exclude CD3+ T cells, CD19+ B cells, CD14+ macrophages, CD34+ eosinophil progenitors, CD123+ dendritic cells and TCR). Lin- cells were then selected for CD127 positivity which stains for all ILCs but not NK cells which are CD127-. Finally, CD117 (c-kit) and CRTh2 (prostaglandin D2 receptor, CD294) markers were used to delineate between the 3 ILC subclasses whereby ILC1 were defined as Lin-CD127+CRTh2-CD117-, ILC2 as Lin-CD127+CRTh2+CD117+ and ILC3 as Lin-CD127+CRTh2-CD117+ cells.

☒ Tick this box to confirm that a figure exemplifying the gating strategy is provided in the Supplementary Information.
